# The mismeasurement of complexity: provider narratives of patients with complex needs in primary care settings

**DOI:** 10.1186/s12939-019-1010-6

**Published:** 2019-07-04

**Authors:** Fiona Webster, Kathleen Rice, Onil Bhattacharyya, Joel Katz, Eric Oosenbrug, Ross Upshur

**Affiliations:** 1 Arthur and Sonia Labatt Family School of Nursing, Faculty of Health Sciences, Western University, 1151 Richmond Street, Room 2373, London, Ontario N6A 3K7 Canada; 2 Department of Family Medicine, McGill University, Montreal, QC Canada; 3 Women’s College Research Institute, 790 Bay St, 7th Floor, Toronto, Ontario M5G 1N8 Canada; 4 Department of Family and Community Medicine, University of Toronto, 500 University Avenue, 5th floor, Toronto, Ontario Canada; 5 Department of Psychology, York University, 4700 Keele St., BSB 232, Toronto, Ontario M3J 1P3 Canada; 6 Clinical Public Health, Dalla Lana School of Public Health, University of Toronto, 155 College Street, Room, Toronto, Ontario 632 Canada

**Keywords:** Complex patients, Institutional ethnography, Chronic pain, Health equity

## Abstract

**Purpose:**

Chronic disease is a global concern. While ample research has aimed to identify the epidemiology of multimorbidity and patient complexity using administrative data, little attention has been paid to the processes of care that treating complex patients entail. Consequently, the concept of patient complexity itself does not directly speak to how challenging it may be to care for a given patient. The purpose of this study was to investigate how primary care providers define, encounter, and manage complex patients, especially those with chronic pain. To our knowledge, this is the first study to move beyond general narrative descriptions of complexity towards an interrogation that is grounded in the work practices of caring for these patients.

**Methods:**

We undertook an institutional ethnography (IE) in Ontario, Canada. IE uses people’s everyday work problems as the starting point for an exploration of the often-invisible social relations that orient experiences. Grounded in the everyday experience of primary care providers, we draw here on 51 interviews that were collected as part of our larger IE study, to interrogate the utility of definitions of patient complexity as medical multimorbidity.

**Findings:**

Care providers consider patients challenging due to their socio-economic status more so than their medical problems alone. Our data shows that patients’ issues are often bound up with poverty, trauma, and mental health concerns, and are challenging for health care providers in part because the interventions needed exceed the scope of their medical expertise, while social issues render the treatment of potentially straightforward medical problems complicated. This was especially so for patients with chronic pain.

**Conclusion:**

Defining patient complexity as morbidity alone is inadequate; such models neglect syndromes and conditions that are not included in formal disease classifications. Chronic pain should be included among the chronic conditions that are considered to constitute multimorbidity. In order to provide effective patient-centered care, discussions of patient complexity must also attend to the complex social and economic circumstances in which many patients live and include broader issues of inequity and social justice. This approach would enable policies to better support primary care providers who struggle to manage their patients with complex needs across domains of physiological health, mental health, and the quality of their living conditions, and in so doing improve the care that patients receive.

## Introduction

Chronic disease is widely recognized as an issue of global concern [1, 2]. Of the growing number of people with chronic conditions, the incidence and prevalence of patients with multimorbidities – that is, patients with more than one concurrent chronic condition – continue to rise [3, 4]. In many countries, this burden is compounded by an aging population and rising life expectancy [5]. Often referred to as complex patients [6, 7], multimorbid patients are likely to be admitted and readmitted to hospital [8, 9], to die prematurely [9], to report low quality of life [9, 10], to suffer from depression and poor mental health [9], and to require costly coordination across specialties [5]. Consequently, complexity and multimorbidity are significant concerns for research [11–13] and policy [14, 15].

To that end, the focus of most relevant research has been on the use of administrative data to identify which patients experience the greatest overall number of morbidities, especially to identify those “complex patients” who are high-cost users of the health care system [e.g. 16–18]. While raw data from these studies indicate that low socio-economic status (SES) is a significant factor in both developing multimorbidity and for heavy use of health care systems, analyses of these data have homed in on the frail elderly as the most complex kind of patient, perhaps because this demographic tends to have the most concurrent chronic conditions relative to other groups [8, 16–18].

Furthermore, while considerable attention has been devoted to identifying the epidemiology of multimorbidity and patient complexity using administrative data, comparatively little attention has been paid to the processes of care that treating complex patients entail, and to identifying what high-quality, patient-centered care for these patients should look like [10]. Consequently, the concept of patient complexity itself does not directly speak to how challenging or straightforward it may be to provide high-quality, patient-centered care. In Canada, this ambiguity is compounded by a narrow definition of multimorbidity that includes 9 common medical conditions yet excludes complex chronic conditions like pain [19, 20], and ignores the well-established relationship between socioeconomic status (SES) and patient capacity to cope with chronic conditions. As Grant et al. note, “caring for an adherent and insured patient with 5 well-controlled comorbid conditions may be relatively straightforward, whereas management of another patient with fewer conditions may present a considerable challenge because of psychosocial or nonmedical factors” [21] p. 797 . In a new series focused on complexity, Greenhalgh et al. noted that RCTs are limited in their ability to answer questions about complexity and called for other research designs, including “powerful ethnographic narratives paying attention to interconnectedness and incorporating an understanding of how systems come together as a whole from different perspectives” [11].

We aimed to identify narratives of patient complexity from the standpoint of primary care health professionals, and to ground our investigation in their accounts of the work that caring for complex patients entails. Drawing on 51 interviews with primary care providers (physicians, nurses, and allied health professionals), our study interrogates the utility of definitions of patient complexity as multimorbidity. While one recent small-scale qualitative study examines definitions and experiences of complexity from the perspective of primary care providers [12, 13], to our knowledge, ours is the first to move beyond general narrative descriptions of complexity towards an interrogation of patient complexity that is grounded in the work practices that caring for these patients involves, and provides a link between the organization of this work and institutional practices, including policies. It is also the first large-scale ethnographic study to examine patient complexity from the standpoint of primary care providers.

## Methods

### Design

This study is part of an ongoing institutional ethnography (IE) aimed at investigating how primary care physicians define, encounter and manage complex patients with chronic pain [22]. As reported elsewhere [22–25], our overarching research question was: How do primary care physicians describe the *work* they do in caring for patients with complex chronic conditions, especially pain? This study included ethnographic observation in primary care, textual analysis, and key informant interviews. This article reports on 51 interviews that were carried out as part of this larger study.

### Data collection

Understanding the social world requires taking up a specific position as a starting point from which to explore how things are put together [26]. In this sense, IE purposively samples an institutional process rather than a population. The goal is not to be representative of the broader population, but rather to understand a phenomenon or process in-depth. Participants (61) were identified by the study team and through a process similar to snowball sampling [see 22], were recruited via scripted email. The first 8 interviews were conducted by EG, a Master’s-trained research assistant, and the remaining interviews and observations were carried out by KR, a post-doctoral medical anthropologist with extensive experience in qualitative data collection in health care settings. FW and KR met to de-brief following every interview and observation. Interviews were guided by a semi-structured interview guide developed by FW, which was structured to emphasize the actual work that people perform including activities not normally considered formal work, such as filling out forms, etc. The interviews began with broad, open-ended questions about the interviewees’ scope of practice, before moving on to discussions of patients with medical complexity. Questions about the work involved in caring for these patients were integrated throughout the interview, while queries that explicitly focused on chronic pain were posed towards the end. The material discussed in this paper is derived from early portions of the interviews, where interviewees spoke more generally about their experiences of caring for patients with complex conditions.

### Analysis

The procedural steps involved in analyzing data are similar to those practiced in other qualitative research (QR) approaches. Interview data were coded prior to analysis. Codes identify features of the data that are pertinent to the research questions and organize data into more concise ideas that can be eventually grouped into topics. Coding of the first few transcripts was performed by FW and KR, who then met to compare codes, notes, and preliminary reflections. Multiple coding is a useful way of developing reflexivity – that is, rather than a tool to confirm the “truth” of the data [27]. Reflexivity used in this way refers to the process by which we critically examine our own assumptions about the world. Through comparison of our respective and evolving understandings of the transcript data, each member of the coding team has an opportunity to check their own assumptions and ideas. The closest equivalent for this type of analytic technique would be thematic analysis [28]. However, codes as they are used in IE are not used to develop themes but instead reflect our analytic interest in explicating how the work that is performed by an actor in one situation (locally) is coordinated extra-locally. Once codes were established, all transcripts were coded, and team meetings were held to analyze the data. Our team included a post-doctoral fellow trained in medical anthropology (KR), two family physicians (OB, RU), a pain psychologist (JK), and a PhD student studying in health policy and health psychology (EO). The diversity of training and experience of our multi-disciplinary team allowed for the practice of reflexivity, through which a singular view or interpretation could never dominate the analysis. During these meetings, we discussed and reflected on our emerging understanding of what was being described. The thoughts and comments that resulted from all meetings were recorded as extensive marginal notes throughout the interviewing, transcription, and coding phases, with the goal of focusing thoughts around the emerging concepts. These meeting notes also become part of the audit trail in which the team keeps careful track of all relevant interpretative and theoretical decisions.

### Data management and steps to ensure quality

All interviews were audiotaped, professionally transcribed, and organized using NVivo software. Ethical approval was gained from the research ethics board of our university prior to data collection. All participants provided informed signed consent to participate.

### Findings

When explicitly asked to define a complex patient, interviewees referred both to patients who were complex from a strictly medical standpoint, and patients whose medical problems were compounded by challenging life circumstances. Indeed, most interviewees drew attention to a distinction between patients whose complexity was *strictly* medical (that is, all of their morbidities fit within recognized medical diagnoses and whose treatments fell within the provider’s scope of practice), and socially complex patients. Regarding the latter, physicians emphasized that the patient’s medical problems could be relatively straightforward in theory, yet in practice be greatly complicated by social circumstances that hindered their ability to manage their medical conditions. The following account is typical of the descriptions of complex patients that were given to us by interviewees:

*I would say a complex patient is someone who has multiple things going on, and often someone who has low capacity to deal with those multiple issues. So, frequently I would say someone who has some physical health issue, some mental health issue, and some social issue, it’s usually their social issue that makes them complex, and it’s all interconnected in what led them to their social issue; that may be the mental health issue, may be the physical health issue, or often social issues beget other social issues, so it’s kind of all mixed in together (Family Physician, Interview #11).*

Physicians frequently made reference to “some social issue” as in the quotation above or to similarly vague adjectives meant to categorize and delineate the “medical” from both mental health and addictions and the broader lives their patients were living. Such vague references highlight how difficult it was for most physicians to understand and articulate their patients’ lives and living circumstances.

When asked for concrete examples of complex patients that they themselves had treated, almost without exception, providers described patients whose lives involved ongoing struggles with poverty, longstanding abuse and trauma, and inadequate mental health care. Consider the following example that was given to us, in response to a request for an example of a patient that would be considered complex:

*The first person who came to my mind, however, medically speaking [she’s] probably not so much [complex], but one that’s come to my mind is a young lady who’s had a complex trauma history, a lot of significant sexual abuse, and physical abuse repeated cumulative across her early childhood into adolescence. She has chronic suicide in her family with several members on a particular side of the family. She has also grown up in a situation where she’s caregiver for complex disability, family members that have osteogenesis imperfecta, so really complex in that those conditions are really unique, and some real specialization around the family care and the family system. That was pretty complex and unique (Counsellor/Therapist at Community Health Centre, Interview 14).*

It also became clear that the complexity of these patients was compounded by a health care system that was poorly coordinated and ill-equipped to provide physicians with the resources necessary to care for their patients. Some perceived that providing good care for complex patients would entail significant transformation of the health care system:

*When we talk about patients being medically complex, part of the problem is the creation of complexity by the actions of health care providers and the system itself, and often you need a truly interprofessional group to kind of articulate what the care goals are, bring them into alignment, and put a care plan in place (…) The thing about complex patients, they’re incredibly time-consuming, and if we’re going to adapt to this broad implementation we need to really fight the time battle. [And] clinicians won’t change the way they book patients, they love to say, you know, “we’ll deal with one problem only” which is the stupidest thing you can do (Primary care physician, Interview#1).*

However, some physicians also differentiated between patients they considered “difficult” and patients who were complex. When asked to expand on this distinction, several physicians described “complex” patients using a medical definition of multi-morbidity but then talked about patients who “frustrate them”.

*A complex patient is one who has different needs, medical needs or psycho-social needs. A challenging patient, I guess, for me, is one who frustrates me in one form or another. An example of challenging patients would be patients who frequently miss appointments, patients who don’t attend any appointments that I arrange for them, specialist appointments or do tests that I request. So, when I think of challenging patients, that’s what I think of. I think it’s completely different from complex patients (Primary care physician, Interview #30).*

In this description, the physician does not consider that perhaps a patient who frequently misses appointments may be someone who may also be dealing with a challenging home or life situation. Finally, unprompted, many primary care physicians recounted stories of dealing with patients with chronic pain when asked to describe patients they considered complex (see also Table 1). One physician noted that:

**Table 1:** Chronic Pain Patients as Complex Patients: Supplementary Quotes

*One of my most complex patients would be an elderly woman who is one of my chronic pain clients. She’s got multiple concerns every time she comes in. She’s got bowel troubles, she’s got blood pressure issues, she’s got cholesterol problems. She’s got lots of chronic pain, and it’s in her neck, in her shoulder, in her hip. She’s got osteoporosis. She’s got eye problems. She’s really attached to me, so it’s really difficult to also get her engaged with other sources here. She comes to me and she likes to tell me about all her different problems and then it means getting her sorted to all the different specialists that she might need. She’ll often go to the specialist and then still not be satisfied with what they had to say and want a second opinion. I find that can be quite complicated (Interview 15, nurse practitioner).*	
*We have a couple of patients coming through actually, who have had chronic pain, fibromyalgia-type diagnosis, and they’ve also had gastric bypass surgery, and then there’s also some previous injuries or surgeries on knees, or whatever it might be. Those are a couple of our complex patients coming through, and they have a difficulty at times, being heard, I think, and being understood. The complexity of the gastric bypass, and how they absorb medications and things like that, is something that they feel that people don’t quite understand, for them. Because they feel once they’ve had the gastric bypass surgery, that there’s no … absorption of the medication is completely different, and it might travel through their system faster or slower, or whatever it might be, and they feel that they’re not being heard in terms of they actually have legitimate pain (Interview 20, nurse).*	
*From a standpoint of true complexity, I had a young woman as an example, a young mother, who developed what’s called chronic regional pain syndrome, used to be RSD, so what I’ve come to think of as a substantial wiring problem in the pain system (Interview 22, Family Physician).*	
*Complex would be a patient … we have a patient that has cancer. I think it’s colon cancer. He is a smoker. He has hypertension and diabetes. He’s obese. He has a lot of pain. So that would be a complex patient just because he’s on chemo and then half the time when he’s walking here he’s in so much pain. He has trouble walking from one end to the other. Like him, it would be trying to make him comfortable and stuff but then his significant other is … she’s an alcoholic and I don’t know if she takes drugs or not but that’s complex too. The whole situation, like you’ve got to look at the patient plus whatever happens with the patient, like things that happen around the patient, so significant other and other stressors (Interview 24, nurse).*

*I consider my complex patients my ones that have lots of medical conditions that you need to manage and multiple medications. My description of a challenging patient is more often behavioural, whether it’s from a psychiatric perspective. It’s just how I define them as challenging. They may have more psychiatric underlying issues or actually often chronic pain (Primary care physician, Interview #21).*

In the above account, references to secondary gain, behavioural issues and depression that manifests as pain link to discourses in which patient concerns and experiences are de-legitimized as they are cast as being drug-seeking, seeking benefit from claiming pain, or suffering psychiatric issues. And again, nowhere in this account does the physician reference or acknowledge the social determinants of health.

Not all clinicians adopt this language however when sharing examples from their practice. One nurse described her understanding of how poverty, chronic pain, and system issues intersect in the life of a patient with “long-term lower finances” who is unable to work due to pain and endures a long wait for surgery related to a lack of specialists locally (Primary care nurse, Interview 18). Another physician was explicit that patients can be complex due to social and structural issues, saying,

*Yeah, I mean I guess we have medically complex patients, we get referred sometimes patients from the hospital who don’t have a family doctor, those tend to be the medically complex patients with multiple medical issues, on a large number of medications. We use the term complex and vulnerable, partially because sometimes patients are complex because of their social circumstances, because they’re under-housed, living in shelters or homeless* (Primary care physician, Interview #5).

The degree of compassion and empathy expressed for these patients also varied. It is important to note that some physicians described liking their patients with complex needs, and found their care rewarding. Nevertheless, this did not change their underlying assumptions. For example:

*I find chronic pain patients really interesting to me (…), you can learn a lot from these patients. But to learn how to manage them, it really takes years. They’re very difficult. So, I find knowing about the chronic pain is very useful in general practice, and knowing the psychiatry of these patients extremely useful, and that’s a huge lack in our healthcare teaching. To see these patients, it takes quite a few years to figure them out and get used to them, and to know what to do with them. And, you have to be extremely patient as well, and a little bit creative. You have to know about all different branches of medicine to some extent, because it’s all going to come into the management (Primary care physician, Interview 38).*

Physician accounts of their patients often discursively located complexity as residing within the patient more so than the system, in many cases ignoring a broader societal context that shaped that inequality. For a small number of physicians, this resulted in them expressing views that were dismissive and even stigmatizing. This perspective is sharply conveyed by a physician who felt that because patients’ complex social needs are outside the scope of biomedical expertise, they should not expect clinicians to resolve their problems:

*These socially-dysfunctional patients, this is the worst group, because they will come over and over, like a boomerang. There is no way to establish rapport, there is no way to establish boundaries because there are no boundaries. They will be most demanding. Some of them have real personality disorders and there’s not much can be done. So, this patient, pretty much in my opinion, they need to be kicked out of the family practice. They actually need to be kicked out of the medical system generally speaking, because they’re not really patients … (Internal Medicine Specialist, Interview 36*).

Some, however, pointed to system issues, pointing out that poor care coordination is failing patients with complex needs:

*So there’s a lack of continuity. The services that people attend are by and large pretty good, but if you’re not very bright, you don’t speak very good English, you’re not very mobile, then you can fall through the cracks and there’s no way for the system to easily pick that up and respond (…). The fact is that it’s a disaster what the system is currently. There is a lot of different strains that have made it a disaster (Primary care physician, Interview 7).*

And, similarly:

*I think the bottom line is that it’s very, very challenging. I think there are a lot of things in our current health care environment that are not sustainable. The transition periods are always the biggest challenge, so from primary care to specialist, from acute hospital to chronic rehab, these transitions, phases. It’s just the way the system, I think, historically has been set up. Patients don’t know how to navigate the system, and we live under this premise that we all have universal access to health care (…) I think there does need to be a lot of changes to the processes of how we manage these patients and help them navigate the system and implement a system that is a little bit more efficient that way (Surgeon, Interview #25).*

## Discussion

Our findings suggest that a definition of patient complexity based solely on presence and number of multimorbidities is far too narrow and does not reflect the local actualities encountered by providers in their everyday work. Indeed, from the perspective of primary care providers, patients who they consider complex are challenging not so much due to their medical problems alone, but rather to their social and living conditions [also 12, 13] as well as their behaviours, which they often described as frustrating. In virtually every example that we were offered, the patient’s medical issues were bound up with poverty, trauma, and mental health concerns. Such patients were challenging for health care providers in part because the interventions that these patients need extend beyond the scope of biomedical expertise, even as their social issues rendered the treatment of potentially straightforward medical problems complicated. The themes of poverty, mental health, and longstanding trauma are remarkably consistent across a range of settings. It is therefore surprising that, until very recently [29], poverty has been largely absent in discussions of complexity and multi-morbidity in Canada. For example, in their retrospective cohort study of all Ontarians, Pefoyo et al. [30] identify a 40% increase in multi-morbidity between 2003 and 2009. They propose a number of possible explanations for this increase, including better tracking of patient data, improvements in medical knowledge and technologies that prolong life, and unhealthy behaviours. Although they identify SES as an indicator of multi-morbidity, they do not consider the impact of social inequality and lack of social supports on the health of Canadians nor in the work of providing care.

While most studies of patient complexity do not explicitly examine SES, our findings are nevertheless supported by recent quantitative analyses of administrative data and thus enrich quantitative research aimed at identifying and describing complex patients. For example, using a web-based tool to categorize 120 randomly-selected patients from a roster in Massachusetts, Grant et al. [21] found that patients deemed complex were more likely to reside in low-income areas, not to have completed high school, and to require psychotherapy. Moreover, they found that among younger patients, complex patients were more likely to be female, to be members of a visible minority, to have had a positive urine test for drug or alcoholuse, to have had a diagnosis of alcoholism or hepatitis C, and to have been prescribed medication for smoking cessation. A rare Canadian study that that analyzed administrative data according to patient SES found that high-cost users of the Ontario health care system are disproportionately female, low-income, food insecure, and likely to have low levels of educational attainment [16]. However, the authors report that their methodologies excluded both individuals who are homeless or members of First Nations living on reserves. Both of these groups are linked with low socio-economic status, and are frequently omitted from large quantitative studies because they are often undocumented in administrative databases. Had these groups been included in the analysis, the association between poverty, educational status, and food security might have been even more pronounced.

When asked for concrete examples of complex patients whom they had treated, a high proportion of care providers offered examples of patients suffering from chronic pain alongside other conditions. This suggests that working definitions of complex patients should be expanded to encompass both SES and chronic pain. This call to include chronic pain in definitions of complexity is also supported by quantitative analyses of administrative data. For example, recent research examining multi-morbidity in primary care using a pan-Canadian electronic medical record database found that the most common clusters of chronic conditions are anxiety and depression, and chronic musculoskeletal problems [31]. This study also found that individuals with these two morbidities represented the vast majority of patients with chronic multi-morbidity. This is concerning given studies most frequently-cited in Canadian policy documents relating to multi-morbidity exclude both mood disorders and musculoskeletal problems among the chronic conditions that they examine, focusing instead on conditions such as hypertension, chronic obstructive pulmonary disease, and congestive heart failure, which affect the elderly disproportionately e.g. [32, 33]. While chronic pain is not considered one of the conditions factored into the medical definition of complexity, our findings lead us to believe that it should. In addition, many physicians in our study did not seem aware of how the social determinants of health might manifest in the behaviours and actions of their patients. Underneath many of the accounts that were provided to us, often couched in biomedical terms, were stereotypes of drug-seeking, benefit-seeking or depressed patients, whose descriptions of their symptoms could not be trusted. As we have noted elsewhere, this introduces into the traditional work of providing care by way of diagnosis and treatment, the burden of determining which patients are truly ill, truly suffering with pain and who might therefore be properly eligible for care [25]. In addition, patient experiences are subjective and the biomedical model is premised on the notion of objectivity. Chronic pain, suffering, poverty, depression and notions of secondary gain are entirely subjective and thus are at odds with the model in which most physicians are trained.

The frail, multimorbid elderly require high-quality coordinated care. However, we argue that poor people with depression, anxiety, and chronic pain are a uniquely significant cohort, both in scale and in the challenge that they pose for primary health care providers. Our study suggests that these patients cannot be ignored even from the most fiscally oriented perspective, as they present an enormous and overwhelming burden for primary care providers. As we have written elsewhere [22, 25] many primary care providers are asked to treat patients whose complexity is grounded in complex social problems that are far beyond the scope of a physician’s time and expertise. With large numbers of physicians choosing career options other than primary care in hopes of avoiding such struggles [34], this should be of concern to policymakers and patients alike.

## Limitations

Interview questions about patient complexity were posed prior to asking explicitly about chronic pain. Nevertheless, participants had received a recruitment email and consent form that made our interest in chronic pain explicit. Their discussions of patient complexity may thus reflect a bias toward having chronic pain patients come to mind. Nevertheless, data suggesting that the majority of multimorbid Ontarians suffer from chronic pain and depression; a heavy representation of chronic pain patients among the examples given may thus be an accurate reflection of interviewees’ experiences treating patients with chronic pain.

## Conclusion

Our study presents rich qualitative data that supports and contextualizes recent quantitative studies that suggest that patient complexity may be more effectively defined in relation to socioeconomic and mental health status, as opposed to medical multimorbidity exclusively. In so doing, we challenge the assumption that complex patients are typically frail and elderly. Such assumptions are widespread in contemporary policy discussions in Canada and abroad, yet are evidently incomplete. While many frail elderly *do* have complex primary care needs, drawing on in-depth interviews with primary care providers we found that the patients they *experience* as complex are complex due to their SES more so than to the nature of their biomedical conditions. This has significant policy implications in that medically complex patients require effective integration of care across medical specialties, while the complex patients from these providers’ narratives suggest many complex patients cannot be healthy without support, services, and interventions that fall beyond the medicalized scope of providers’ clinical training. In order to provide effective patient-centered care, discussions of patient complexity must attend to the complex social and economic circumstances in which many patients live and include broader issues of inequity and social justice.

Furthermore, when asked for concrete examples of patients they considered complex, a large proportion of interviewees offered examples of patients who struggled with chronic pain alongside other biomedical and social conditions. Supported by evidence that chronic pain is prevalent among individuals with multimorbidity, we argue that chronic pain should be included among the conditions that are considered to define multimorbidity. This approach would enable policies to better support primary care providers who struggle to manage their patients with complex needs across domains of physiological health, mental health, and the quality of their living conditions, and in doing so improve the care that patients receive.

## Data Availability

The datasets generated and/or analyzed during the current study are not publicly available due to the nature of qualitative research in which anonymity and confidentiality is assured during the research process but additional excerpts are available from the corresponding author on reasonable request.
